# The Oral–Gut Microbiome–Brain Axis in Cognition

**DOI:** 10.3390/microorganisms13040814

**Published:** 2025-04-03

**Authors:** Noorul Ain Adil, Christabel Omo-Erigbe, Hariom Yadav, Shalini Jain

**Affiliations:** 1 USF Center for Microbiome Research, Microbiomes Institute, Tampa, FL 33612, USA; nadil@usf.edu (N.A.A.); omoerigbe@usf.edu (C.O.-E.); hyadav@usf.edu (H.Y.); 2 Department of Neurosurgery and Brain Repair, University of South Florida, Tampa, FL 33612, USA

**Keywords:** oral microbiome, cognition, brain, inflammation, dysbiosis

## Abstract

Alzheimer’s disease (AD) is a progressive neurodegenerative disorder characterized by cognitive decline and neuronal loss, affecting millions worldwide. Emerging evidence highlights the oral microbiome—a complex ecosystem of bacteria, fungi, viruses, and protozoa as a significant factor in cognitive health. Dysbiosis of the oral microbiome contributes to systemic inflammation, disrupts the blood–brain barrier, and promotes neuroinflammation, processes increasingly implicated in the pathogenesis of AD. This review examines the mechanisms linking oral microbiome dysbiosis to cognitive decline through the oral–brain and oral–gut–brain axis. These interconnected pathways enable bidirectional communication between the oral cavity, gut, and brain via neural, immune, and endocrine signaling. Oral pathogens, such as *Porphyromonas gingivalis*, along with virulence factors, including lipopolysaccharides (LPS) and gingipains, contribute to neuroinflammation, while metabolic byproducts, such as short-chain fatty acids (SCFAs) and peptidoglycans, further exacerbate systemic immune activation. Additionally, this review explores the influence of external factors, including diet, pH balance, medication use, smoking, alcohol consumption, and oral hygiene, on oral microbial diversity and stability, highlighting their role in shaping cognitive outcomes. The dynamic interplay between the oral and gut microbiomes reinforces the importance of microbial homeostasis in preserving systemic and neurological health. The interventions, including probiotics, prebiotics, and dietary modifications, offer promising strategies to support cognitive function and reduce the risk of neurodegenerative diseases, such as AD, by maintaining a diverse microbiome. Future longitudinal research is needed to identify the long-term impact of oral microbiome dysbiosis on cognition.

## 1. Introduction

Cognitive impairment refers to a significant decline in mental abilities that affects daily living activities. This term encompasses both mild cognitive impairment (MCI) and dementia, which together contribute to progressive cognitive decline [1,2]. MCI represents an early stage of cognitive deterioration, progressing to dementia at an annual rate of 5–10%, with prevalence rising from approximately 6% in individuals aged 60–64 to around 25% in those aged 80–84 [3]. Individuals with MCI are at high risk of further progression to more severe cognitive impairment [4]. Alzheimer’s disease (AD), the most prevalent neurodegenerative disorder among the elderly, is characterized by a gradual decline in cognitive and behavioral functions, including memory, comprehension, language, attention, reasoning, and judgement. AD affects approximately 57 million people worldwide, with a mortality rate of 35% within 5 years of diagnosis [5]. Before the COVID-19 pandemic, AD was the sixth leading cause of death in the United States, and it ranked seventh after the pandemic [2]. By 2050, the number of people affected by AD is expected to rise to 152 million [6]. Elucidating the key factors influencing cognitive function, particularly during early stages, like MCI, is critical.

The oral and gut microbiomes are essential components of the human microbiota, each playing a vital role in health by influencing immune function, metabolism, and disease susceptibility through intricate microbial interactions and systemic pathways [7]. The oral microbiome comprises a complex community of microorganisms, including bacteria, fungi, protozoa, and viruses [8]. This diverse ecosystem contributes to physiological processes, such as digestion, pathogen defense, and oral homeostasis. Conversely, the gut microbiome consists of microorganisms residing in the gastrointestinal tract, primarily the intestines, and is instrumental in nutrient absorption, immune modulation, and the synthesis of essential vitamins and neurotransmitters [9]. Together, the oral and gut microbiomes form a dynamic and interconnected system that influences metabolic functions, immune responses, and overall health [10].

Over the past decade, research exploring the relationship between the microbiome, particularly the gut and oral microbiomes, and neurodegenerative diseases has expanded significantly [11,12]. This growing body of evidence has identified opportunities for preventive care and potential therapeutic strategies targeting AD. While the gut microbiome has been extensively studied in relation to neurodegeneration, the oral microbiome remains comparatively underexplored, despite its direct exposure to environmental pathogens and systemic interactions [13,14]. Growing research indicates that changes in the composition of the oral microbiome, particularly the presence of certain bacteria, like *Porphyromonas gingivalis*, may be linked to an increased risk of developing AD [15]. The oral–brain and oral–gut–brain axis illustrate the complex mechanisms by which the oral immune system interacts with the brain through systemic inflammation and immune signaling [16]. These pathways, mediated by neural, immune, and endocrine systems, including the vagus and trigeminal nerves, demonstrate how oral microbial homeostasis contributes to systemic and neurological stability [17,18]. Dysbiosis within the oral or gut microbiomes can induce systemic inflammation, disrupt brain function, and contribute to neurodegenerative processes [11,12]. Additionally, dysbiosis within these microbiomes has been associated with systemic diseases, such as diabetes, cardiovascular disease, and metabolic syndrome, all of which also contribute to cognitive decline [19]. Given these associations, an integrated healthcare approach that addresses both oral and gut microbiome health is essential to support neurological well-being. This review examines the influence of the oral microbiome on cognitive function, emphasizing the key factors that shape its composition and the consequences of microbial dysbiosis. Additionally, it investigates the interplay between oral and gut microbiome imbalances and their combined effects on cognitive decline [20].

## 2. Oral Microbiome and Cognitive Impairment 

The oral microbiome significantly influences cognitive health due to its extensive and diverse microbial ecosystem [21]. Estimated species diversity in the oral microbiome vary, with some studies suggesting over 700 species [14]. Whilst other reports estimated 50 to 100 billion bacteria present in the average adult oral cavity, encompassing roughly 200 dominant bacterial species. In total, around 700 predominant taxa have been identified within the oral microbiome. Notably, less than one-third of these taxa have been successfully cultured in vitro, highlighting the challenges of fully characterizing the microbial diversity in the oral cavity [22]. Additionally, the oral microbiome is not limited to bacteria; it also includes fungi, viruses, and protozoa [23]. The oral microbiome is the second largest microbial community in the human body, second only to the gut [24]. These microbes inhabit various hard and soft tissues within the oral cavity, such as teeth, oral mucosa, cheeks, tonsils, gums, saliva, and both hard and soft palates [25]. The oral microbiome is integral to host–microbiome interactions in the oral cavity. Although core microbiomes are considerably consistent across individuals but still variations occur per individual due to several environmental and genetic factors.

The oral microbiome thrives under specific conditions, including an average temperature of 37 °C and decreasing bicarbonate (HCO_3_^−^) and hydrogen ions, making a saliva pH range of 6.5–7 [26], although some studies suggest a broad range of 6.2–7.6, with an average of 6.7 [27]. Gases, such as carbon dioxide, ammonia, and oxygen, play crucial roles in shaping the oral microbiome by influencing pH levels and overall composition of the oral microbiome. The oral cavity maintains a higher CO_2_ pressure of (27–54 mmHg) compared to the atmospheric air (0.3 mmHg), creating an environment where CO_2_ diffuses out of saliva when exposed to air. This effect is more pronounced during activities, like eating and breathing, promoting a more alkaline environment [28]. In addition to this natural process, ammonia production by the oral bacteria from sources, like arginine and urea, helps further neutralize acids, contributing to a more alkaline environment. Higher ammonia-producing bacteria are associated with a lower risk of caries, as they counteract acid buildup caused by microbial metabolism [29]. Oxygen levels vary significantly within oral biofilms, shaping microbial community composition. Initial colonizers on oral surfaces are exposed to higher oxygen levels, whereas oxygen is limited in mature biofilms due to restricted diffusion. This creates a gradient that supports the growth of aerobic, facultative anaerobic, and obligate anaerobic bacteria. Oxygen also influences sugar metabolism, acid production, stress tolerance, and bacterial survival within dental plaque by altering redox environments [30]. Common bacterial genera found in a stable oral microbiome include *Abiotrophia*, *Peptostreptococcus*, *Streptococcus*, *Stomatococcus*, *Actinomyces* and many more, as further explained in Table 1. Additionally, non-bacterial residents, like protozoa (*Entamoeba gingivalis*) and *Trichomonas tenax*, fungi (*Candida*), and numerous viruses are also prevalent in the oral cavity [31]. 

Disruptions in this symbiotic balance can lead to the overgrowth of pathogenic species, such as *P. gingivalis* and *F. nucleatum*, resulting in oral infections and diseases. The oral microbiome typically exists as a biofilm, which is crucial for oral microbial homeostasis and protecting against disease development [41,53]. Therefore, ensuring a balanced oral microbiome is not only vital for microbiome stability but also essential for overall systematic well-being including cognitive function. Emerging research indicates that oral microbes and their byproducts can enter the bloodstream, potentially triggering systemic inflammatory responses and affecting various organs [54]. This connection is increasingly supported by studies linking oral microbiota to chronic diseases, such as Alzheimer’s disease, cancer, diabetes, preterm birth, and inflammatory bowel disease [14,55].

Studies have shown mechanisms through which oral infections contribute to systemic inflammation. Inflammatory cytokines and mediators originating from the oral cavity can disseminate to other parts of the body, intensifying systemic inflammation responses [56]. Metabolites produced by oral bacteria, such as lipopolysaccharides (LPS), short-chain fatty acids (SCFAs), peptidoglycans, and hydrogen sulfide (H_2_S) [57]. LPS, a key component of Gram-negative bacterial membranes (*P. gingivalis*, *F. nucleatum*, *A. actinomycetemcomitans*, *T. forsythia*, and *T. denticola*), is a potent driver of systemic inflammation [58]. It binds to Toll-like receptor 4 (TLR4), activating immune cells, such as neutrophils, macrophages, and monocytes [59]. This interaction produces inflammatory cytokines, like IL-6, TNF-α, and IL-1β, contributing to metabolic endotoxemia, insulin resistance, and atherosclerosis. LPS can also cross the blood–brain barrier, activating immune cells involved in neuroinflammation and Alzheimer’s disease [60,61]. Additionally, it influences immune responses in dental stem cells through the NF-κB and Wnt/β-catenin pathways, further underscoring its role in inflammation [62,63].

SCFAs, produced primarily by carbohydrate fermentation from bacteria, such as *Streptococcus*, *Actinomyces*, and *Lactobacillus*, exhibit anti-inflammatory and context-dependent effects. They suppress TNF-α and IL-12 production in the macrophages and dendritic cells, reducing excessive immune responses and promoting the differentiation of regulatory T cells (Tregs), which help maintain immune homeostasis [64,65,66]. SCFAs also help support the intestinal barrier, preventing harmful bacteria from entering the bloodstream and causing inflammation [67]. However, when the gut microbiome becomes imbalanced, harmful bacteria increase and produce endotoxins, like LPS, damaging intestinal cells, weakening the intestinal barrier, and triggering systemic inflammation by activating immune cells [68]. Additionally, in certain conditions, such as obesity and metabolic disorders, excessive SCFAs may contribute to chronic inflammation [69]. SCFAs in the oral cavity are mainly produced through carbohydrate fermentation and amino acid breakdown. In the first process, bacteria, like *Streptococcus* and *Lactobacillus*, break down carbohydrates into sugars, which are then converted into SCFAs [70]. Bacteria can also use salivary mucins to produce SCFAs once broken down. In the second process, bacteria, like *Actinomyces* and *Fusobacterium*, break down proteins into amino acids, which are then processed to produce SCFAs [71]. The levels of SCFAs in the oral cavity can vary, affecting bacterial activity and overall oral microbiome stability [72]. A study observed that AD patients had lower levels of *Akkermansia muciniphila*, aSCFA-producing bacterium, along with reduced propionic acid in both fecal and blood samples, and further investigation in AD model mice and cultured hippocampal neuronal cells revealed that oral propionate supplementation ameliorated cognitive impairment [73]. Dental and gingival health issues, such as periodontal disease, have been linked to an increased risk of cognitive impairment, potentially through the systemic spread of inflammatory markers or harmful bacteria that reach the brain [74].

Peptidoglycans (PGs), found in Gram-positive and Gram-negative bacteria, act as strong immunostimulatory molecules. In Gram-positive bacteria, PGs are detected by TLR2, triggering inflammation, while fragments, like muramyl dipeptide (MDP), are recognized by NOD2, activating NF-κB and cytokine production [75]. Fragments, such asMDP, further trigger inflammasome activation. Studies in TLR2-deficient mice have shown that losing PG recognition increases susceptibility to infections, like *Streptococcus pneumoniae* and *Staphylococcus aureus* [76]. In Gram-negative bacteria, PG fragments and LPS are recognized by PRRs, like NOD1, NOD2, TLR4, TLR2, MD-2, and CD14, triggering immune responses [77]. Systemically, PGs have been linked to gut microbiome dysbiosis, rheumatoid arthritis, and other autoimmune conditions [78]. Periodontitis secretes bacterial pathogens, like LPS and peptidoglycans, modifiable risk factors for AD, and has been linked to dementia risk through systemic inflammation [79].

Studies suggest a potential link between periodontitis—a severe form of gum disease—and cognitive decline. Oral pathogens and their byproducts can potentially infiltrate the central nervous system through the nerves or blood vessels, affecting the brain tissues susceptible to neurodegenerative processes [80]. Periodontitis-associated pathogens, including Gram-negative bacteria, like *Porphyromonas gingivalis*, *Treponema denticola*, *Fusobacterium nucleatum*, *Prevotella intermedia*, and *Aggregatibacter actinomycetemcomitans*, as well as Gram-positive bacteria, like *Streptococcus* spp. and *A. meyeri*, can contribute to AD risk by infiltrating the central nervous system (CNS) through systemic circulation and neural pathways [15,81]. These bacteria release virulence factors, like LPS, gingipains, and peptidoglycans, which can cross the blood–brain barrier, and trigger neuroinflammation and amyloid plaque formation [81,82]. Additionally, metabolic byproducts, such as SCFAs (propionate, and acetate) and hydrogen sulfide (H_2_S), produced by dysbiosis of the oral microbiota, may exacerbate neurodegenerative processes by promoting oxidative stress and neuroinflammation [83]. A study found that transplanting saliva from periodontitis patients into mice via oral gavage induced intestinal dysbiosis and inflammation, suggesting that periodontitis can contribute to these conditions through the influx of salivary microbes [84].

Longitudinal studies support this association, showing that factors such as the severity of periodontitis, tooth loss, and oral hygiene practices correlate with cognitive decline and the risk of Alzheimer’s disease. For instance, a 20-year study involving 152 participants between the ages of 50 and 70 showed an inverse relationship between the severity of periodontitis and cognitive function [85]. A 2019 retrospective cohort study utilizing National Health Insurance data found that individuals with chronic periodontitis had a 95% higher risk of developing dementia, regardless of otherwise healthy lifestyle habits [86]. A literature review of English language publications from 2018 to 2022 was conducted, analyzing data from PubMed, Cochrane, Scopus, and Web of Science. The findings highlight a significant link between periodontitis and Alzheimer’s disease, particularly among elderly individuals with additional risk factors and poor oral health conditions [86,87]. Another study with a 32-year span and 597 male participants indicated that tooth loss, periodontal pocket depth, and alveolar bone loss were associated with cognitive impairment, especially in those that were over the age of 45 [88]. Additionally, a cohort study with 144 subjects found that having the Apolipoprotein E. (APOE-ε4) gene and fewer teeth were linked to accelerated cognitive decline. The study population consisted of Catholic nuns with relatively uniform socioeconomic backgrounds, as 85% held a bachelor’s degree or higher and 88% were teachers [85]. Their shared living conditions and access to an on-site dental clinic minimized socioeconomic disparities, allowing for a more controlled examination of the relationship between oral health and cognitive function [89]. The polymorphism in Apolipoprotein E (APOE) is characterized by three major isoforms, ε2, ε3, and ε4, which differ by single amino acid substitutions, influencing lipid metabolism and neuronal health [90]. Among these, the APOE-ε4 isoform is strongly linked to cognitive decline, with impairments in memory, executive function, and increased seizure risk, likely due to early amyloid accumulation [91]. The ε4 isoform promotes amyloid accumulation by reducing amyloid-beta (Aβ) clearance and enhancing its aggregation, leading to increased Aβ plaque deposition in the brain [92,93]. APOE-ε4-positive elderly individuals perform worse on tasks involving object recognition and spatial memory, and the isoform is associated with earlier dementia onset and a higher risk of epilepsy following brain injury. These cognitive differences may be due to accumulated pathology over time, which impairs performance in older APOE-ε4 carriers compared to non-carriers [94].

Other studies have highlighted the correlation between dental health habits and cognitive impairment. For example, a follow-up survey involving 5468 subjects revealed that irregular tooth brushing habits were associated with an increased risk of AD [55]. Another survey of 2355 U.S. adults aged 60 and older, accounting for race/ethnicity (non-Hispanic White, non-Hispanic Black, Mexican–American), income level (≤$14,999, $15,000–24,999, >$25,000), health insurance status (private, Medicare, Medicaid, military), education level (<12 years, 12 years, >12 years), and smoking status (never, current, former), found an association between periodontitis and cognitive impairment [95]. A study analyzing 605 participants aged 60–69 found that greater oral microbial α-diversity was significantly correlated with higher cognitive performance DANTES Subject Standardized Tests (DSST) and a reduced likelihood of subjective memory decline, suggesting a potential link between oral microbiome diversity and cognitive health [96]. All these studies suggest that the oral microbiome significantly influences cognitive function.

## 3. Oral Microbiome and Gut Microbiome

The human microbiome consists of trillions of microorganisms that inhabit various parts of the body, including the oral cavity and the gut [97]. The oral microbiome and gut microbiome are two distinct yet interconnected ecosystems that play crucial roles in systemic well-being. Factors such as diet, genetics, environment, and lifestyle influence the composition of these microbiomes [98]. Disruptions in these microbial communities can contribute to a range of health conditions, highlighting the need to elucidate their interactions and regulatory mechanisms. Recent advancements in metagenomics and bioinformatics have provided deeper insights into these microbial communities at the species level [99]. This knowledge is pivotal for developing targeted therapies to restore microbial balance and improve health outcomes. Investigating how the oral and gut microbiomes influence each other can uncover new pathways for disease prevention and management.

The oral microbiome begins to form during or even before birth when the fetus is exposed to amniotic fluid. While the microbial load in the oral cavity is initially low, its composition gradually develops, influenced by factors such as maternal transmission, breastfeeding, and environmental exposure [14,100]. Before the eruption of teeth, microorganisms, such as *Streptococcus*, *Actinomyces*, and *Lactobacillus*, are dominant [49,101]. Once the teeth begin to erupt, typically after the first year, colonization by periodontal microbes leads to the development of plaque [102]. This process introduces the oral cavity to a more diverse set of microorganisms, establishing the core oral microbiome, which may influence the risk of oral diseases later in life. These findings highlight the importance of high diversity of microorganisms in the oral microbiome from an early age to promote systemic well-being [103].

**Figure 1:**
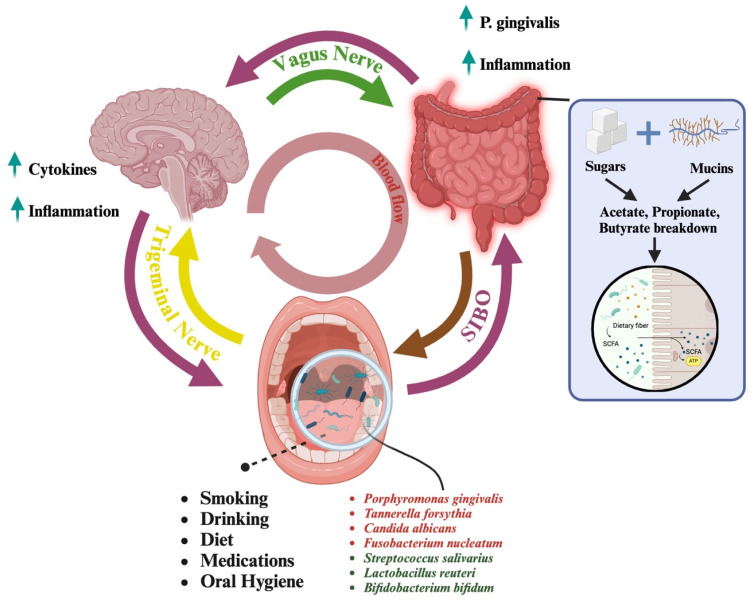
An overview of the pathways connecting the oral microbiome to the brain highlights two distinct mechanisms of interaction. The first pathway is the oral-to-gut-to-brain axis (pink arrows). In this pathway, bacteria from the oral cavity travel to the gut, where certain prominent microorganisms, such as *Porphyromonas gingivalis*, can induce inflammation. This inflammation plays a critical role in the gut-brain connection, which is facilitated by the vagus nerve (green arrow). The relationship between the oral cavity, gut, and brain is bidirectional, allowing for continuous communication and mutual influence among these systems. Additionally, conditions, such as small intestinal bacterial overgrowth (SIBO), can facilitate the reverse movement of bacteria from the gut back to the oral cavity (brown arrow). In cases of SIBO, excessive bacterial growth in the small intestine can lead to reflux of gut bacteria into the oral cavity, disrupting the balance of the oral microbiome and further contributing to systemic inflammation. Connected to the gut is the conversion of sugars and mucins to SCFAs (in the blue box). Dietary sugars and host-derived mucins are fermented by gut microbes into short-chain fatty acids (SCFAs), like acetate, propionate, and butyrate. These SCFAs support host health by providing energy, strengthening gut integrity, and regulating immunity. The second pathway is the direct oral-to-brain connection, which is facilitated by the trigeminal nerve (yellow arrow). This pathway enables microorganisms from the oral cavity to directly impact brain health. Notably, this connection is also bidirectional, allowing the brain to signal and regulate the activity of specific microorganisms through the lymphatic system. Together, these pathways illustrate the complex and dynamic interaction between the oral microbiome and the brain, emphasizing the connection of microbial, gut, and neural health. Within the oral cavity, the presence of various microorganisms can be influenced by factors such as smoking, alcohol consumption, diet, medication, and oral hygiene practices. Some microorganisms have detrimental effects (in red) on the oral microbiome, while others contribute positively to maintaining oral health. Microorganisms with harmful effects are often associated with behaviors or conditions that disrupt the balance of the microbiome, while beneficial microorganisms thrive in environments (in green) supported by proper oral hygiene and healthy lifestyle choices. Created in https://BioRender.com, accessed on 24 March 2025.

The gut microbiome is distributed throughout the entire gastrointestinal tract and, most extensively studied in the colon and large intestine, where microbial density is highest [104,105]. While bacteria are present along the gastrointestinal tract, short-chain fatty acid (SCFA)-producing bacteria are predominantly found in the colon, where they ferment dietary fibers to generate SCFAs, such as butyrate, acetate, and propionate, which play crucial roles in maintaining gut health and modulating brain function [106]. Along with playing a role in numerous physiological processes, a study suggests gut microbiota interacts intricately with host metabolism [107]. They found that certain gut bacteria, such as *Bacteroides* and *Firmicutes*, are crucial in fermenting dietary fibers, producing SCFAs that promote gut health and metabolic homeostasis. SCFAs, like butyrate, propionate, and acetate, reduce pro-inflammatory cytokine production by binding to GPR41 and GPR43 receptors on immune cells, helping to control inflammation [108]. These SCFAs promote the differentiation of regulatory T-cells (Tregs), which further aid in immune regulation and prevent excessive inflammation [67]. Butyrate supports the gut barrier by nourishing colonocytes, reducing immune activation by preventing harmful pathogens from entering the bloodstream [109,110]. Additionally, SCFAs contribute to the high diversity of microorganisms in the gut microbiome, influencing immune cell activity and cytokine production and regulating systemic immune responses [110]. Studies have shown distinct changes in the SCFA levels in AD patients, with SCFAs contributing to the improvement of AD-related cognitive impairment [111]. SCFAs, particularly acetate, propionate, and butyrate, are crucial in mitigating cognitive impairment in AD. For instance, clinical studies have shown that higher serum concentrations of acetate and valerate correlate with elevated Aβ levels, while butyrate levels are inversely related to Aβ concentrations [111]. SCFAs are produced by gut bacteria that ferment indigestible dietary fibers and modulate microglial activation, inhibiting neuroinflammation [112]. In AD, SCFAs, especially butyrate, help improve cognition by reducing peripheral inflammation and enhancing blood–brain barrier (BBB) integrity. They also influence microglial activity, decreasing neuroinflammation [113], which is crucial for mitigating cognitive decline. Furthermore, SCFAs can elevate the expression of brain-derived neurotrophic factor (BDNF), a key gene for neurogenesis and synaptic plasticity, further enhancing cognitive function [114]. These effects, along with the ability of SCFAs to restore microbial balance and protect the BBB, suggest their therapeutic potential in AD management [111]. Additionally, research explored the gut microbiome’s role in modulating the gut–brain axis, showing how imbalances in the microbiome can influence neurological conditions, such as AD, through inflammation, SCFA production, and blood–brain barrier integrity [115]. Gut microbiome dysbiosis, contribute to neurological disorders, like AD, by inducing neuroinflammation, altering microglial activation, disrupting immune signaling through chemokines and cytokines, and affecting the production of neurotransmitters and neuroactive compounds, like SCFAs, which are essential for CNS homeostasis [116]. The gut–brain axis is a bidirectional communication pathway that involves neural, hormonal, and immune mechanisms, further highlighting the systemic impact of the gut microbiome, which is illustrated in Figure 1.

Strategies to enhance microbiome diversity, such as probiotics, prebiotics, and dietary modifications, are increasingly recognized for their potential to support cognitive function [106]. Probiotics and prebiotics have also been linked to cognitive function enhancement in healthy individuals, with supplementation for four to six months improving memory, attention, and visuospatial abilities in aging populations [117]. Sodium oligomannate (GV-971) has also shown promise in reversing cognitive impairment, correcting gut dysbiosis, and reducing neuroinflammation while penetrating the blood–brain barrier to inhibit Aβ fibril formation [118]. Liu et al. (2021) found that 5XFAD mice treated with mannan oligosaccharide showed reduced cognitive deficits, fewer amyloid plaques, and lower oxidative stress and microglial activation [119]. Periodontitis and IBD are chronic inflammatory diseases linked through the oral–gut axis, where *P. gingivalis* translocates to the gut, disrupts microbiota, and promotes inflammation, while oral-derived Th17 cells migrate to the intestine, interact with translocated *P. gingivalis*, and exacerbate colitis through immune activation and increased IL-1β levels [120]. A recent mouse study showed that oral administration of *P. gingivalis* induced intestinal dysbiosis, compromised barrier function, and triggered inflammation, while gut microbiota transplantation further clarified its role in disrupting intestinal homeostasis [84]. The interconnectedness of the oral microbiome with the gut microbiome is further supported by a study by Li et al., who demonstrated that the oral microbiome is capable of modifying the gut microbiome and altering its composition [101,107]. Excessive invasion of the oral microbes into the gut microbiome has been observed in patients diagnosed with inflammatory bowel disease, where oral commensal microbes, such as *Haemophilus* and *Veillonella*, have been found within the gut mucosa [101,121]. The interconnection of oral and gut microbiomes has a significant impact on cognitive function.

## 4. Oral to Gut Transfer

The oral microbiome is a gateway to the gut microbiome. Studies have revealed that approximately 45% of bacterial species found in the gut originate from the oral cavity, underscoring the significant contribution of oral bacteria to gut microbial diversity [13]. A considerable portion of the 55% of gut bacteria originates from diet and food sources, but the gut microbiota is also influenced by the intestinal barrier, which combines physical, biochemical, and immunological factors to protect the host and regulates microbial interactions, enzymes, and glycosylation patterns in the gut mucus further shape microbial colonization, affecting diversity and functionality [122]. The host immune system, including antimicrobial peptides and secretory IgA, helps maintain balance by controlling bacterial overgrowth and supporting beneficial species. Environmental factors, like geography, lifestyle, and antibiotic use, can disrupt microbiota, emphasizing the critical balance between host and microbes for overall diversity [105].

Saliva microbes suggest that *P. gingivalis*, a known pathogen associated with periodontitis, can influence the gut microbiome due to its pathogenic properties [123]. Gingipains (proteolytic enzymes) and lipopolysaccharides (LPS) are key virulence factors of *P. gingivalis*, a major pathogen in periodontitis, that enable it to influence systemic health [124]. *P. gingivalis* and its outer membrane vesicles (OMVs) evade immune detection and infiltrate distant organs. In the brain, its LPS and gingipains disrupt the blood–brain barrier (BBB), triggering neuroinflammation and promoting amyloid-beta accumulation, a hallmark of Alzheimer’s disease [125].

The presence of *P. gingivalis* in the gut has the potential to disrupt the normal balance of the gut microbiota, leading to a range of adverse effects. Once in the gut, this bacterium is able to withstand the acidic conditions and proliferate, resulting in dysbiosis [126]. This dysbiosis can contribute to the development of gastrointestinal disorders, such as inflammatory bowel disease (IBD), colorectal cancer, and even metabolic conditions, like obesity and diabetes [19]. Additionally, *P. gingivalis* in the gut may influence systemic inflammation, contributing to the progression of diseases beyond the gastrointestinal tract, such as cardiovascular diseases and neurodegenerative disorders, like AD [127]. As a result, the oral–gut microbial axis plays a crucial role in modulating immune responses and metabolic health. Studies have demonstrated that oral microbiota influence gut-associated lymphoid tissue (GALT) by regulating dendritic cell activity, T-cell differentiation, and cytokine production, ultimately shaping systemic immunity [128,129]. Moreover, alterations in oral bacterial populations have been linked to an increased risk of metabolic syndrome and obesity, likely due to their effects on gut microbiome composition, systemic inflammation, and insulin sensitivity.

Dysbiosis in the oral microbiome, characterized by an overgrowth of pathogenic bacteria, contributes to metabolic dysfunction [24]. *P*. *gingivalis* produces LPS that enter circulation, triggering low-grade systemic inflammation, insulin resistance, and increased gut permeability (“leaky gut”), all of which promote metabolic diseases, like type 2 diabetes and obesity [130]. Similarly, *Fusobacterium nucleatum*, a pathogen associated with periodontal disease, can translocate to the gut, disrupting gut microbiota composition and impairing immune and metabolic regulation [131]. Additionally, *Treponema denticola*, a spirochete linked to periodontitis, secretes virulence factors that exacerbate chronic inflammation, further increasing the risk of obesity and type 2 diabetes [132]. The overgrowth of pathogenic bacteria (*P. gingivalis*, *F. nucleatum*, *T. denticola*) and the resulting inflammatory and metabolic disruptions have been associated with an approximately 30% increased risk of developing metabolic syndrome and obesity [133]. These findings underscore the importance of high diversity of microorganisms in the oral microbiome for optimal gut health and overall well-being along with the significance of microbial transfer from the oral cavity to the gut in metabolic regulation.

Saliva contains antimicrobial components that regulate microbial populations while also serving as a vector for bacterial transmission to the gut during ingestion, facilitating the introduction of commensal bacteria that contribute to gut microbial diversity [134,135]. Moreover, oral bacteria produce metabolites and other compounds that help to strengthen the mucosal lining of the gastrointestinal tract, such as *Streptococcus salivarius* (*S. salivarius*) and *Streptococcus mitis* (*S. mitis*), which also restrain the growth of pathogenic and harmful bacteria [136]. This reinforcement of the mucosal barrier further strengthens the intestinal barrier. As *S. salivarius* and *S. mitis* contribute to the production of bacteriocins [137], these antimicrobial peptides exhibit activity against various pathogens. Salivaricin A and Salivaricin B, produced by *S. salivarius*, integrate into bacterial membranes and create pores, leading to leakage of cellular contents and cell death [138]. This mechanism is particularly effective against Gram-positive bacteria, such as *Streptococcus pyogenes* and *Streptococcus pneumoniae* [139]. Additionally, bacteriocins share properties similar to antibiotics, inhibiting bacterial growth by disrupting cell wall synthesis, leading to bacterial lysis. This mechanism is particularly effective against *Haemophilus influenzae* and *Fusobacterium nucleatum* [140]. By exerting these antimicrobial effects, bacteriocins contribute to enhancing the effectiveness of the mucosal barrier, thereby preventing the entry of harmful pathogens into the bloodstream [141]. By limiting the translocation of pathogens, toxins, and pro-inflammatory molecules, a strengthened mucosal barrier helps preserve gut homeostasis and overall digestive health [68,142]. Enhancing gut health through mucosal barrier reinforcement may provide benefits beyond the gastrointestinal tract, particularly for individuals with Alzheimer’s disease (AD) and cognitive impairments. A well-functioning gut barrier can help reduce chronic inflammation and regulate the gut–brain axis, potentially mitigating neuroinflammatory processes associated with AD and other neurodegenerative conditions [143].

## 5. Factors Affecting the Oral Microbiome 

### 5.1. The pH of the Oral Cavity 

The pH level in the oral cavity significantly influences the composition and behavior of the oral microbiome. A neutral to slightly alkaline pH fosters an optimal microbial environment by promoting the growth of alkali-generating commensal bacteria, such as *Streptococcus sanguinis* and *Streptococcus gordonii*, which utilize the urease and arginine deiminase systems (ADS) to neutralize acids and prevent pH-dependent dysbiosis [144]. This pH range supports salivary buffering mechanisms, enhances the activity of antimicrobial peptides (AMPs), and facilitates enamel remineralization by maintaining calcium and phosphate ion availability. Conversely, an acidic environment (pH < 5.5) favors the proliferation of acidogenic and aciduric bacteria, such as *Streptococcus mutans* and *Lactobacillus* spp., *S. mutans* ferments dietary carbohydrates into lactic acid, leading to a local decline in pH [145,146]. The resulting acidic conditions not only promote enamel demineralization but also selectively favor the growth of acid-tolerant bacteria, further reinforcing a cariogenic biofilm. Sustained acidity suppresses beneficial bacteria, impairs host immune defenses, and drives biofilm shifts, increasing the risk of dental caries and periodontal disease [102,147]. Periodontal pathogens and their metabolites can enter the bloodstream, triggering chronic low-grade inflammation, which is a known risk factor for cognitive decline and neurodegenerative diseases, including Alzheimer’s disease [148]. Studies suggest that oral dysbiosis-induced inflammation disrupts the blood–brain barrier, facilitates the accumulation of amyloid-beta plaques, and exacerbates neuroinflammation, all of which are key mechanisms underlying cognitive impairment. Research indicates that when the pH drops below the critical threshold of 5.5, demineralization of tooth enamel accelerates, leading to increased caries risk [149]. Furthermore, fluctuations in pH can also influence the overall stability of the oral microbial community, potentially leading to dysbiosis and oral diseases, such as periodontitis [147]. Therefore, managing oral pH through diet, oral hygiene, and professional care is vital for preventing disease, promoting a diverse oral microbiome, and supporting cognitive function.

### 5.2. Diet

Diet, particularly the intake of sugars and carbohydrates, plays a pivotal role in shaping the oral microbiome, influencing dental health, and cognitive function. Sugars and fermentable carbohydrates serve as substrates for acidogenic bacteria, like *Streptococcus mutans*, which metabolize these compounds into acids. This acid production lowers the pH in the oral environment, leading to demineralization of tooth enamel and promoting dental caries [150]. Research has consistently shown that frequent consumption of sugary and starchy foods increases the risk of dental caries by providing a constant source of energy for these cariogenic bacteria [151,152,153]. Inflammatory mediators and bacterial byproducts, such as *P. gingivalis* lipopolysaccharides (LPS), can enter the bloodstream, disrupt the blood–brain barrier, and contribute to neuroinflammation—an established factor in cognitive decline and Alzheimer’s disease [148]. Managing dietary sugar and carbohydrate intake is therefore critical not only for preventing dental caries but also for supporting the high diversity of microorganisms in the oral microbiome and preventing inflammatory oral conditions.

Vegetarian diets are often associated with a more diverse oral microbiome. Plant-based diets are rich in fibers, vitamins, and minerals that promote the growth of beneficial bacteria in the oral microbiome, including oral Streptococci, such as *Streptococcus sanguinis*, *Streptococcus gordonii*, and *Streptococcus salivarius*, which are some of the first colonizers within the oral cavity [154]. These bacteria contribute to oral health by supporting pH homeostasis, inhibiting the proliferation of pathogenic species, and facilitating nitrate reduction, which promotes nitric oxide production and vascular health [155]. Additionally, dietary fibers stimulate salivary flow, enhancing the natural cleansing mechanism of the oral cavity and further supporting a diverse microbial community. High fiber content from vegetables and fruits stimulates saliva production, which helps maintain a diverse oral microbial environment by washing away food particles and buffering acids produced by bacteria. This buffering action helps stabilize the pH of the oral cavity, as discussed previously [13]. On the other hand, diets heavy in meat can alter the oral microbiome in ways that may increase the risk of dental diseases. High protein and fat intake from meat can create a more acidic oral environment, which can promote the growth of harmful bacteria associated with periodontal disease and caries [79]. However, meat also provides essential nutrients, like iron and zinc, which play critical roles in immune function, wound healing, and cellular metabolism. These nutrients contribute to the maintenance and repair of oral tissues, supporting periodontal health and resistance to infection. When consumed in moderation with fiber-rich plant foods, meat can contribute to a nutritionally diverse diet that supports both microbial homeostasis and overall systemic health [156]. Therefore, balancing diet and food intake is a key component for neutralizing the oral microbiome.

### 5.3. Medications

The widespread use of medications for managing chronic conditions has a profound impact on the oral microbiome, often altering its delicate balance. Drugs, such as antihypertensives, antidiabetics, and antibiotics, can disrupt the microbial diversity and composition within the oral cavity [157]. These changes may arise from side effects, like dry mouth, immune modulation, or shifts in metabolic function, which create conditions that favor the growth of pathogenic bacteria over beneficial species [158]. The prevalence of these medications and their effects on the oral microbiome is crucial, as they can increase susceptibility to oral diseases, such as periodontal infections, dental caries, and oral candidiasis. Recognizing these risks highlights the importance of mitigating adverse effects through targeted interventions, such as saliva stimulants or probiotic therapies, to preserve oral microbiome stability while addressing systemic conditions [13]. Since the oral microbiome plays a crucial role in modulating systemic inflammation and maintaining a balanced gut microbiome, disruptions caused by chronic medication use may have downstream effects on brain health. Inflammation originating from dysbiosis in the oral cavity has been associated with neurodegenerative conditions, such as Alzheimer’s disease, highlighting the importance of mitigating these adverse effects through targeted interventions, like saliva stimulants or probiotic therapies [159].

Medications commonly used to manage chronic conditions can have unintended consequences on oral microbiome diversity and function by altering the delicate balance of the oral microbiome. Lisinopril, a widely prescribed angiotensin-converting enzyme inhibitor for hypertension, is one such example, often associated with dry mouth (xerostomia), which can contribute to oral diseases, such as dental caries and periodontal disease. Reduced saliva production compromises the microbiome’s ability to prevent dysbiosis, as a result this shift increases the risk of dental caries, periodontal disease, and opportunistic infections, like fungal overgrowths [160]. Similarly, metformin, a first-line treatment for type 2 diabetes, impacts the oral and gut microbiomes indirectly by modulating systemic inflammation and metabolic markers [160]. Diabetic patients often exhibit an altered oral microbiome dominated by pathogenic species, such as *Porphyromonas gingivalis* and *Tannerella forsythia*, further exacerbated by chronic hyperglycemia [161]. While metformin helps regulate blood glucose levels, its influence on oral microbial diversity remains an area requiring deeper investigation. Similarly, hypothyroidism results in a decrease in microbial diversity in the oral cavity. However, studies have shown that the intake of thyroid hormones can result in the decrease of microbial population, such as, *Bacillus*, *Johnsonella*, *Stenotrophomonas*, *Saprospiraceae*, *Actinobacilllus*, and *Mycoplasma* [143].

Semaglutide, a GLP-1 receptor agonist commonly prescribed for weight management and diabetes, may influence oral microbiome diversity and function through its effects on systemic inflammation and metabolic balance. Although direct research on its impact on the oral microbiome is limited, the condition it primarily targets (obesity) is well-documented to disrupt microbial balance within the oral cavity [24]. Obesity-related changes often result in a decrease in beneficial microbial species and an increase in pro-inflammatory bacteria, which can elevate the risk of periodontal disease and other oral conditions [162,163]. By supporting weight loss and improving metabolic health, semaglutide may help restore microbial balance indirectly. This reduction in inflammation and overall improvement in health markers could create a more favorable environment for a diverse and stable oral microbiome community, highlighting its potential benefits beyond weight management [164].

Antibiotics, while essential for combating bacterial infections, have a profound and often disruptive effect on the oral microbiome. These medications work by targeting bacterial pathogens, but their broad-spectrum nature can also eliminate beneficial bacteria that help prevent dysbiosis [165]. This disruption can result in a temporary state of dysbiosis, where pathogenic microorganisms, like *Candida albicans*, can overgrow, leading to conditions such as oral thrush [166]. Additionally, prolonged or frequent antibiotic use can reduce the diversity of the oral microbiome, weakening its resilience and ability to recover from external insults [167]. This diminished diversity may increase susceptibility to opportunistic infections and chronic conditions, such as periodontitis and dental caries. Moreover, the overuse of antibiotics contributes to the development of antibiotic-resistant bacteria, which poses a significant challenge to managing oral infections effectively [168]. To mitigate these effects, the judicious use of antibiotics and the incorporation of probiotics or dietary interventions may help restore and prevent dysbiosis of the oral microbiome [167]. Disruptions in the oral microbiome can create an inflammatory cascade, which has been associated with neurodegenerative diseases through the gut–brain axis [116]. Given that periodontal disease is associated with an increased risk of Alzheimer’s disease, strategies to mitigate antibiotic-induced dysbiosis such as probiotics and dietary interventions may have implications beyond oral health, potentially protecting cognitive function as well [116].

Aging populations, particularly individuals over 50, are commonly prescribed multiple medications that impact the oral microbiome [169]. Drugs, such as antihypertensives, antidepressants, and anticholinergics, often cause dry mouth, a condition that reduces microbial diversity and promotes pathogenic bacteria. This can increase susceptibility to oral diseases, such as periodontitis and caries. Dietary changes, like incorporating celery or sugar-free lozenges into the diet, can stimulate saliva production, helping restore the microbiome’s balance [170]. Addressing dry mouth in aging populations is critical for microbial homeostasis for both oral health and cognitive well-being, as oral inflammation has been linked to neurodegenerative conditions, which is further discussed in the mechanism of oral–brain and oral–gut–brain section [148].

### 5.4. Smoking 

Smoking is known to alter the oral microbiome, increasing the risk of periodontal diseases. It can reduce the diversity of the microbiome and favor the growth of harmful bacterial species, such as *Actinomyces* and *Veillonella* species [171,172]. Smoking is a well-known risk factor that disrupts this microbial ecosystem, thereby affecting the oral microbiome [173]. The relationship between smoking and the oral microbiome involves changes in microbial diversity and abundance, immune response modulation, and increased susceptibility to oral diseases [174].

Smokers exhibit a less diverse microbiome compared to non-smokers, which may reduce the oral cavity’s ability to resist pathogenic infections [175]. The decrease in microbial diversity is a result of two potential reasons: the change in pH and the exposure to chemicals through smoking [172]. Cigarette smoke contains a complex mixture of chemicals, including toxic compounds, such as nicotine, tar, and carbon monoxide [37,176]. These chemicals can have bactericidal or bacteriostatic effects on microbial species. Smoking can alter the pH of the oral cavity, making it more acidic due to the production of acids in the smoke and decreased saliva production. A lower pH can inhibit the growth of bacteria that thrive in neutral or slightly alkaline conditions, thus contributing to a less diverse microbiome [177]. Smoking further exacerbates this imbalance by promoting the growth of acidogenic and anaerobic bacteria, such as *Fusobacterium*, *Campylobacter*, and *Tannerella forsythia*, which are associated with periodontal disease and systemic inflammation [175]. Additionally, smoking fosters an environment conducive to *Actinomyces* and *Veillonella* species, which, as previously discussed, contribute to biofilm formation and acidification of the oral cavity, further disrupting microbial homeostasis and increasing disease susceptibility [172].

In smokers, specific shifts in microbial composition have been observed. Saliva is enriched with *Treponema* and *Selenomonas*, while the tongue is enriched with *Dialister* and *Atopobium* [178]. These bacterial groups are associated with periodontal diseases and inflammation. Bleeding Index (BI) values measuring gum inflammation show complex microbial correlations. BI values are negatively associated with *Cardiobacterium* in saliva and *Granulicatella* on the tongue, suggesting these species might have protective roles in oral microbiome diversity [179]. In contrast, BI values are positively associated with pro-inflammatory species, like *Treponema*, *Oribacterium*, *Dialister*, *Filifactor*, *Veillonella*, and *Selenomonas* in saliva and *Dialister*, *Bifidobacterium*, *Megasphaera*, *Mitsuokella*, and *Cryptobacterium* on the tongue [180].

### 5.5. Drinking 

Alcohol, as a common dietary component, can influence this microbial environment. The interactions between alcohol consumption and the oral microbiome is crucial for comprehending its broader health implications [181]. Alcohol has inherent antimicrobial properties that can alter microbial balance by inhibiting the growth of certain bacteria while allowing others to proliferate. This selective pressure can lead to dysbiosis [182]. Alcohol disrupts the balance of the oral microbiome by encouraging the proliferation of pro-inflammatory bacteria, including *Neisseria*, *Actinomyces*, *Aggregatibacter*, *Kingella*, *Leptotrichia*, *Cardiobacterium*, *Bacteroidales* [G-2], and *Prevotella*, while diminishing beneficial commensals, like *Lactobacillus*, amongst many more discussed in Table 2. These microbial shifts contribute to the development of dental caries, periodontal disease, and endocarditis [183]. Ethanol metabolism in the oral mucosal microbiome leads to the production of acetaldehyde, a genotoxic compound that damages DNA in epithelial cells by forming harmful DNA adducts, further disrupting microbial homeostasis [184]. Alcohol facilitates the translocation of LPS across the gut, leading to significantly elevated circulating LPS levels in individuals with alcohol-related liver mimics bacterial infection, triggering acute inflammation. Elevated systemic LPS levels trigger an immune response, increasing the production of pro-inflammatory cytokines which have been implicated in neuroinflammation and cognitive decline [60]. Alcohol consumption can reduce saliva production, leading to a dry mouth. Saliva is essential for washing away food particles and microbes, buffering oral pH, and providing antimicrobial proteins [185,186]. Reduced salivary flow compromises these protective mechanisms, facilitating overgrowth of pathogenic microbes mentioned earlier as pro-inflammatory bacteria. An increase in alcohol consumption potentially lowers the pH level, which inhibits the growth of acidophilic bacteria further enhancing the risk of dental erosion and caries [187].

### 5.6. Oral Hygiene 

Chronic inflammation of the tissues supporting the teeth, or periodontal disease, has been connected to systemic inflammation and is thought to be a possible risk factor for several illnesses, including Alzheimer’s disease [194,195,196]. One proposed mechanism involves the release of pro-inflammatory cytokines, such as IL-6 and TNF-α, which can enter the bloodstream and cross the blood–brain barrier, triggering neuroinflammation. Additionally, *Porphyromonas gingivalis* and its toxic byproducts, including gingipains, have been detected in the brains of Alzheimer’s patients, where they contribute to tau protein hyperphosphorylation and neuronal dysfunction, further linking periodontal disease to cognitive decline [194]. The mechanism is further discussed in the section of “Mechanism of Oral–Brain Axis”. However, overusing oral hygiene products can disrupt the delicate equilibrium of the oral microbiome. Excessive use of antiseptic mouthwashes and toothpastes with strong antibacterial agents can eliminate not only harmful bacteria but also beneficial microorganisms [190]. These beneficial bacteria, such as *Streptococcus salivarius* and certain *Lactobacillus* species, play a crucial role in inhibiting the growth of pathogenic microbes through competitive exclusion and the production of antimicrobial compounds. Overusing antiseptic mouthwashes more than twice a day can significantly reduce microbial diversity in the oral microbiome, leading to an overgrowth of opportunistic pathogens, like *Candida albicans* and *Fusobacterium nucleatum*. This imbalance increases susceptibility to dental and gingival health issues such as infections, oral thrush, and bad breath [188].

Balancing oral hygiene practices is thus critical for preserving the beneficial aspects of the oral microbiome. Using oral hygiene products in moderation and selecting those with a balanced antimicrobial spectrum can help maintain microbial diversity [197]. For instance, mouthwashes containing natural antimicrobial agents, like xylitol and essential oils, have been shown to effectively reduce harmful bacteria without significantly disrupting beneficial microbial communities. A study demonstrated that mouthwashes with essential oils could reduce plaque and gingivitis while preserving the diversity of the oral microbiome, highlighting their potential as a balanced alternative to more aggressive antiseptics [198]. Mouthwashes containing essential oils, such as thymol, eucalyptol, menthol, and methyl salicylate, have been shown to reduce plaque and gingivitis effectively. Additionally, lemongrass essential oil-based mouthwashes have exhibited antimicrobial and anti-biofilm activities against oral pathogens, including *Streptococcus mutans* and *Lactobacillus acidophilus* [199].

A recent study, “Children Tooth Brushing Behavior and Oral Microbiota: A Pilot Study”, examined the relationship between tooth brushing frequency and the composition of oral microbiota in children. The study found that brushing twice a day was associated with a healthier oral microbiota profile, reducing harmful bacteria linked to dental caries. The frequency of brushing affects the diversity and abundance of oral bacteria, emphasizing the importance of regular brushing for preventing microbial dysregulation and preventing dental diseases [200]. Individuals with dysphagia often experience poor oral hygiene, creating an environment that promotes bacterial growth and increases the prevalence of pathogenic oral bacteria. Studies have shown that those with dysphagia tend to have greater food debris accumulation, increased plaque buildup, reduced oral clearance, and more frequent denture-related issues [201,202,203].

Additionally, incorporating probiotics into oral hygiene routines is emerging as a promising approach to support a diverse oral microbiome. Probiotic lozenges and mouthwashes containing beneficial bacterial strains, such as *Lactobacillus reuteri* and *Bifidobacterium bifidum*, can help restore microbial balance and promote oral microbiome diversity and function [204]. A randomized controlled trial found that participants using probiotic lozenges experienced a significant reduction in gingival inflammation and an increase in beneficial bacterial species compared to the control group [191,192].

## 6. Mechanism of the Oral–Brain Axis

The complex ways in which the immune system of the oral cavity interacts with the brain through systemic pathways of inflammation and immune signaling emphasize the crucial role played by the oral microbiome [205]. The diverse population of microbes in the oral cavity is essential for harmonious oral microbiome. However, when there is an occurrence of dysbiosis, infections, like periodontitis, can develop. These infections trigger an immune response, leading to a release of pro-inflammatory cytokines, like interleukin-1 beta (IL-1β), interleukin-6 (IL-6), and tumor necrosis factor-alpha (TNF-α) [79,206]. These cytokines then enter the bloodstream and induce systemic inflammation, affecting distant organs, including the brain [207]. These mediators can cross the blood–brain barrier, triggering neuroinflammatory processes that may contribute to neurodegeneration [196]. Additionally, oral pathogens, such as *Porphyromonas gingivalis* and their toxic byproducts, including gingipains, have been detected in the brains of individuals with Alzheimer’s disease, further supporting the potential mechanistic link between periodontal inflammation and cognitive decline [206]. These gingipains, specifically arginine-gingipain (Rgp) and lysine-gingipain (Kgp), have been detected in the brains of Alzheimer’s disease patients. Their presence correlates with pathological markers, such as tau protein tangles and ubiquitin accumulation, which are characteristic of Alzheimer’s pathology [208,209]. Gingipains contribute to neurodegeneration by promoting tau protein hyperphosphorylation and disrupting neuronal function [210]. Additionally, *P. gingivalis* can release outer membrane vesicles containing gingipains and other virulence factors, facilitating their entry into the brain and exacerbating neuroinflammatory processes [211]. These findings suggest a mechanistic link between periodontal disease and Alzheimer’s disease, highlighting the potential role of gingipains in the disease’s progression [125].

The blood–brain barrier (BBB), which typically protects the brain from harmful substances, can be compromised by these pro-inflammatory cytokines. IL-1β, IL-6, and TNF-α disrupt the tight junction proteins that maintain BBB integrity, allowing these cytokines to penetrate the brain parenchyma [80,212]. Once in the brain, these cytokines activate microglia, the resident immune cells, leading to further cytokine production and reactive oxygen species (ROS) release, perpetuating a cycle of neuroinflammation [213]. Chronic inflammation is known to contribute to several neurological disorders, including Alzheimer’s disease. Research has shown elevated levels of cytokines, like IL-6, in the brains of Alzheimer’s patients, correlating with disease severity [214,215]. More studies have found a direct connection between periodontal disease and an increased risk of Alzheimer’s disease [216]. Pathogens, such as *P. gingivalis*, and their virulence factors, such as gingipains, have been detected in the brains of Alzheimer’s patients, indicating either direct microbial invasion or an inflammatory response indirectly contributing to neurodegeneration [217,218]. Evidence underscores the importance of the microbiome-axis, demonstrating that systemic inflammation due to oral dysbiosis can have a significant impact on cognitive function.

Moreover, the presence of lipopolysaccharides (LPS), a potent endotoxin produced by Gram-negative oral pathogens, such as *P. gingivalis*, has been shown to further exacerbate neurodegenerative pathways [219]. Beyond compromising the BBB, LPS directly interferes with synaptic transmission by disrupting long-term potentiation (LTP) a fundamental mechanism underpinning learning and memory [220,221]. LPS-induced impairments in LTP have been associated with decreased synaptic plasticity, ultimately impairing cognitive function and contributing to the hallmark memory deficits seen in Alzheimer’s disease [222]. Additionally, chronic LPS exposure sustains microglial activation, perpetuating cycles of oxidative stress and inflammatory signaling that amplify neuronal injury [223].

Emerging research also suggests that the impact of oral dysbiosis on the brain extends beyond traditional inflammatory pathways, implicating microbial proteins in the direct misfolding of neural proteins [224]. Certain oral bacteria, including *Streptococcus mutans* and *Actinomyces* spp., secrete amyloidogenic proteins that closely resemble human amyloid-beta (Aβ) peptides in structure [225]. Through a process known as molecular mimicry, these bacterial amyloids may act as templates, seeding the aggregation of endogenous Aβ within the brain. This not only accelerates the formation of insoluble amyloid plaques but may also prime the brain’s innate immune system, particularly microglia, to mount exaggerated inflammatory responses against perceived threats, compounding neuronal damage [226,227]. Over time, this cross-reactivity between microbial and host amyloids fosters a chronic state of neuroinflammation and synaptic loss, both of which are central to the progression of cognitive decline.

## 7. Mechanism of the Oral–Gut–Brain Axis

The neural pathways connect the central nervous system (CNS) and the enteric nervous system (ENS), linking the oral cavity, gut, and brain through complex communication networks [228]. The vagus nerve, a critical component of the parasympathetic nervous system, extends from the brainstem to various organs, including the gastrointestinal (GI) tract, and facilitates bidirectional communication between the gut and the brain by transmitting sensory information from the gut to the brain and motor signals from the brain to the gut [229,230]. For example, taste receptors in the oral cavity can activate the vagus nerve, which influences gut motility and secretion [231].

The ENS, often referred to as the “second brain”, consists of a dense network of neurons embedded in the GI tract [232]. It operates independently of the CNS but communicates frequently with it, processing signals from the oral cavity and the gut, coordinating digestive processes, and sending feedback to the brain [233,234]. This system can influence emotional and cognitive functions, as gut-derived signals reach the brain via vagal and spinal pathways, affecting mood and behavior [235,236]. The trigeminal nerve, or fifth cranial nerve, plays an essential role in transmitting sensory information from the oral cavity to the brain, and this connection is profoundly influenced by the oral microbiome [237]. This process begins when sensory receptors in the oral cavity detect stimuli. The trigeminal nerve comprises three branches: the ophthalmic, maxillary, and mandibular nerves, with the latter two innervating the oral cavity to detect stimuli like temperature, pain, touch, and pressure. Once these sensory receptors detect stimuli, they send signals through the trigeminal nerve fibers to the trigeminal ganglion, a cluster of nerve cell bodies near the brainstem [238]. From the trigeminal ganglion, sensory information is transmitted to the brainstem’s trigeminal sensory nuclei, where it is initially processed. This process facilitates the perception of various oral stimuli, such as pain and temperature [239]. The processed information is then relayed to higher brain centers, including the thalamus and cerebral cortex, for comprehensive integration and response [240,241].

The oral microbiome’s ecosystem directly impacts this neural pathway. Disruptions in this microbiome, such as an overgrowth of pathogenic bacteria, can lead to infections and inflammation, activating the trigeminal nerve [242]. Pathogenic bacteria release toxins and inflammatory molecules that stimulate sensory receptors linked to the trigeminal nerve, sending signals of pain and discomfort to the brain [239]. This can result in conditions, like dental pain or trigeminal neuralgia [243,244]. Furthermore, chronic oral infections and inflammation can have systemic effects, impacting both the oral microbiome and overall well-being [245]. Inflammatory mediators released in the oral cavity can enter the bloodstream, affecting distant organs, including the brain [246]. Evidence suggests that chronic oral infections may contribute to neuroinflammatory conditions, impacting brain function and potentially leading to neurodegenerative diseases [247,248]. Thus, the trigeminal nerve not only connects the oral cavity to the brain regarding sensory information but also plays a critical role in the interaction between the oral microbiome and systemic health [249,250].

Oral dysbiosis influences gut function via modulation of enteric glial cells (EGCs), which maintain intestinal barrier integrity [251]. Dysbiotic oral bacteria, once translocated to the gut, can disrupt EGC homeostasis, impairing tight junction protein expression and increasing gut permeability, which in turn elevates systemic endotoxin levels and promotes neuroinflammation [252]. Furthermore, certain oral pathogens, such as *Fusobacterium nucleatum*, have been shown to disrupt the mucosal immune system of the gut by promoting dendritic cell maturation and skewing T-cell responses toward a pro-inflammatory Th17 phenotype, thereby exacerbating chronic intestinal inflammation that can amplify neuroimmune signaling and contribute to cognitive dysfunction [123,253].

The immune system plays a central role in the oral–gut–brain axis through its interactions with microbial populations and signaling pathways. The oral cavity hosts a diverse and dynamic microbiome crucial for maintaining oral and systemic health [14]. These microbial imbalances can enter the bloodstream and travel to the gut, impacting the gut microbiome and local immune responses [254]. Oral bacteria, such as *Porphyromonas gingivalis*, can exacerbate gut issues and alter the gut microbiota composition [255], underscoring the systemic influence of oral health on gut health. The gut microbiome is home to trillions of microorganisms that play a crucial role in digestion, immune health, and brain function. These microbes produce metabolites, such as short-chain fatty acids (SCFAs), which have systemic effects and can influence brain function [114]. Studies have shown that gut microbiota can affect the permeability of the intestinal barrier, a condition known as “leaky gut”, allowing molecules to enter the bloodstream and reach the brain, impacting overall health and contributing to various bodily processes [122,256]. The effect of specific bacteria is further discussed in Table 3.

## 8. Area of Further Research

Significant progress has been made in uncovering the intricate relationships between the oral and gut microbiomes and their influence on systemic health, yet many aspects require further investigation. Research into the specific interactions between the oral and gut viromes, as well as the molecular pathways regulating microbial–host dynamics, will be critical for advancing targeted interventions to mitigate conditions such as Alzheimer’s disease. Identifying how probiotics, prebiotics, vitamins, and dietary modifications shape microbial diversity, regulate immune signaling, and reduce systemic inflammation is a promising avenue for therapeutic development.

Comparing the microbiome composition and functional products between healthy individuals and those affected by neurodegenerative diseases can provide valuable insights into disease mechanisms. Longitudinal studies tracking microbiome dynamics over time in healthy individuals and neurodegenerative patients are essential. These studies can reveal temporal changes in microbial composition, identify early biomarkers of cognitive decline, and assess the efficacy of microbiome-targeted interventions in preserving cognitive function. Additionally, exploring the functional pathway and impact on the oral microbiome with the consumption of medications is needed. Research shows that there is a depletion in the diversity of populations within the oral microbiome, however, there is no major distinction between each medication and its effect.

Future research should prioritize longitudinal studies to examine how chronic medication use influences the oral microbiome over extended periods. Determining the point at which microbial imbalances become clinically significant could enable earlier interventions. Additionally, exploring personalized strategies for preserving microbiome stability based on an individual’s medication regimen is essential. Investigations should assess whether interventions such as artificial saliva, probiotics, or dietary modifications can counteract medication-induced dysbiosis. Comparative studies on different antihypertensive drug classes, such as ACE inhibitors versus beta-blockers, could offer insights into their varying effects on the oral microbiome. Likewise, deeper research into the long-term impact of metformin on oral microbial diversity in diabetic and non-diabetic populations and its impact on cognitive function is necessary. Further research is needed to examine the influence of socioeconomic factors on the relationship between periodontitis, cognitive impairment, and gut health, as existing studies remain limited in these areas.

In the context of thyroid disorders, studies should evaluate how hormone therapy affects microbial populations and overall systemic health, leading to improved management strategies. Since research on GLP-1 receptor agonists and their direct influence on the oral microbiome is limited, clinical trials should be conducted to assess their impact on the oral microbiome and cognitive function. Understanding whether metabolic improvements from weight loss contribute to oral microbial balance could lead to novel therapeutic approaches. Additionally, exploring whether semaglutide reduces oral inflammation associated with periodontal disease may reveal indirect benefits beyond metabolic regulation.

The long-term resilience of the oral microbiome following antibiotic exposure remains an important area of study. Investigating the role of prebiotics, probiotics, and postbiotics in restoring microbial balance after antibiotic treatment could lead to effective therapeutic strategies. Research is also needed to explore the relationship between antibiotic-induced dysbiosis and neuroinflammation, particularly in Alzheimer’s disease models, to better understand the systemic consequences of oral microbiome disruptions. Additionally, evaluating the effects of different antibiotic classes on microbial diversity—particularly whether narrow-spectrum antibiotics pose a lower risk of dysbiosis—could guide more targeted and responsible prescribing practices. Pinpointing specific bacteria that positively or negatively impact cognition could lead to targeted therapies.

Aging populations, often subjected to polypharmacy, experience significant shifts in microbial homeostasis. Future studies should investigate how medication-induced oral dysbiosis contributes to cognitive decline and neurodegenerative diseases. Research into dietary and lifestyle interventions to counteract these microbiome shifts could inform preventive healthcare strategies. Assessing the effectiveness of specific foods or supplements in alleviating dry mouth-related dysbiosis may lead to practical recommendations for older individuals.

Ongoing studies should aim to understand how medication-induced alterations in the oral microbiome impact dentistry and cognitive function. Research should examine the role of microbial shifts in the development of conditions, such as periodontitis, dental caries, and oral candidiasis. Additionally, understanding how these changes influence restorative dentistry, implant success, and post-surgical wound healing could enhance clinical outcomes. Studies on how dental professionals can mitigate medication-related dysbiosis through preventive care, probiotic applications, and saliva-enhancing treatments would provide valuable insights into improving patient care.

## 9. Conclusions

The intricate relationship between the oral microbiome, gut microbiome, and cognitive function underscores their collective influence on the development and progression of neurodegenerative diseases, such as Alzheimer’s disease (AD). The oral–brain axis highlights the direct effects of oral health on neurocognitive function, where oral dysbiosis can trigger systemic inflammation and exacerbate neurodegenerative processes. The detection of oral pathogens, such as *P. gingivalis*, in the brain further emphasizes the critical role of supporting a stable and diverse oral microbiome ecosystem to mitigate cognitive decline. Additionally, the oral–gut–brain axis reveals the interconnected nature of oral and systemic health, as oral bacteria can alter gut microbiota composition, leading to gut dysbiosis. This imbalance impacts systemic inflammation, further influencing cognitive function. Lifestyle factors, such as diet, pH balance, smoking, and alcohol consumption, are pivotal in shaping the oral microbiome, reinforcing the need for comprehensive strategies to improve a well-regulated microbiome for both systemic and cognitive well-being.

Future research should aim to elucidate the specific mechanisms through which the oral and gut microbiota contribute to neurodegenerative diseases. Exploring therapeutic interventions, including probiotics, prebiotics, and targeted dietary modifications, offers promising avenues for restoring microbial balance and mitigating cognitive decline. Advancing knowledge of the oral-brain and oral-gut-brain axes will drive the development of innovative prevention and treatment strategies for Alzheimer’s disease and other neurodegenerative conditions, ultimately enhancing quality of life for at-risk individuals.

## Figures and Tables

**Table 1:** Common bacteria found in the oral cavity.

Microorganism	Type of Bacteria	Increase/Decrease	References
*Bifidobacterium*	Gram positive	Increase: Consume fermented foods (yogurt, kefir), prebiotic fibers (bananas, onions). Decrease: Antibiotic overuse, high-fat diets.	[32]
*Corynebacterium*	Gram positive	Increase: Maintain good hygiene, balanced diet. Decrease: Overuse of harsh skin/oral antiseptics.	[33]
*Eubacterium*	Gram positive	Increase: High-fiber diet, whole grains. Decrease: Processed food consumption, antibiotics.	[34]
*Lactobacillus*	Gram positive	Increase: Consume probiotics (kimchi, miso, sauerkraut). Decrease: Antibiotics, excessive alcohol.	[35]
*Propionibacterium*	Gram positive	Increase: Probiotic-rich foods, fermented dairy. Decrease: Poor hygiene, overuse of antiseptics.	[36]
*Pseudoramibacter*	Gram positive	Increase: Maintain oral health, consume fiber-rich foods. Decrease: Smoking, high sugar intake.	[37,38]
*Rothia*	Gram positive	Increase: Good oral hygiene, balanced diet. Decrease: Poor oral care, excessive sugar intake.	[39]
*Moraxella*	Gram negative	Increase: Exposure to diverse environments, balanced diet. Decrease: Air pollution, antibiotic overuse.	[40]
*Neisseria*	Gram negative	Increase: Balanced diet, oral hygiene. Decrease: Poor dental care, smoking.	[41,42]
*Veillonella*	Gram negative	Increase: Prebiotic foods, high-fiber diet. Decrease: Processed foods, high sugar intake.	[41,42]
*Campylobacter*	Gram negative	Increase: Proper food handling, diverse diet. Decrease: Poor hygiene, contaminated food consumption.	[43]
*Capnocytophaga*	Gram negative	Increase: Maintain good oral health. Decrease: Smoking, high sugar diet.	[41,44]
*Desulfobacter*	Gram negative	Increase: Fermented foods, fiber intake. Decrease: High-fat diet, processed foods.	[45]
*Desulfovibrio*	Gram negative	Increase: Fiber-rich diet, plant-based foods. Decrease: Excessive meat consumption, smoking.	[45]
*Eikenella*	Gram negative	Increase: Good oral hygiene. Decrease: Smoking, excessive alcohol consumption.	[13,41,46]
*Haemophilus*	Gram negative	Increase: Proper respiratory hygiene. Decrease: Smoking, pollution exposure.	[47]
*Leptotrichia*	Gram negative	Increase: Good oral and gut health practices. Decrease: Processed foods, poor dental hygiene.	[31,48]
*Prevotella*	Gram negative	Increase: Whole grains, fiber-rich diet. Decrease: Low-fiber diets, excessive processed food.	[48]
*Selemonas*	Gram negative	Increase: Healthy oral microbiome, fiber intake. Decrease: Excessive sugar, poor oral hygiene.	[49]
*Simonsiella*	Gram negative	Increase: Balanced oral care, diverse diet. Decrease: Overuse of antiseptics, smoking.	[50]
*Treponema*	Gram negative	Increase: Maintain oral health. Decrease: High-sugar diets, smoking.	[51]
*Wolinella*	Gram negative	Increase: Probiotic-rich foods, fiber intake. Decrease: Processed foods, antibiotics.	[52]

**Table 2:** Impact of most common oral microorganisms: sources, pathogenicity, and impact on cognitive functions.

Microorganism	Source	Impact on Cognitive Function	Reference
**Pathogenic**			
*Porphyromonas gingivalis*	Poor diet, Diabetes (hyperglycemia), Periodontitis	*P. gingivalis* releases lipopolysaccharides (LPS) and gingipains, which can cross the blood–brain barrier (BBB) and trigger neuroinflammation. Gingipains contribute to tau protein hyperphosphorylation and amyloid-beta accumulation, both of which are hallmarks of Alzheimer’s disease.	[123,161]
*Tannerella forsythia*	Diabetes (hyperglycemia)	Chronic inflammation driven by *T. forsythia* has been implicated in vascular dysfunction, which may exacerbate neurodegenerative processes. Its role in periodontitis has been linked to elevated systemic inflammatory markers, increasing the risk of cognitive decline.	[161]
*Candida albicans*	Overuse of antiseptic mouthwash, Excessive use of Antibiotics	*C. albicans* has been detected in the brains of Alzheimer’s patients and has been shown to induce beta-amyloid aggregation, oxidative stress, and neurotoxicity. This fungal pathogen has been implicated in cognitive dysfunction, memory deficits, and neuropsychiatric disorders, such as schizophrenia.	[188]
*Fusobacterium nucleatum*	Overuse of antiseptic mouthwash	*F. nucleatum* contributes to neuroinflammation and may accelerate cognitive decline in Alzheimer’s disease. Its involvement in systemic inflammation has been linked to increased brain oxidative stress and neuronal dysfunction, impairing memory and cognitive flexibility.	[188]
*Aggregatibacter actinomycetemcomitans*	Found in dental plaque, especially in individuals with aggressive periodontitis.	Elevated levels of *A. actinomycetemcomitans* have been associated with vascular inflammation, which can compromise cerebral blood flow and contribute to cognitive impairment. Studies indicate that its presence may increase the risk of neurodegenerative conditions by exacerbating systemic inflammatory responses.	[189]
**Beneficial**			
*Streptococcus salivarius*	Healthy oral cavity	*S. salivarius* produces bacteriocins, such as salivaricin, which inhibit the growth of pathogenic bacteria in the oral cavity and gut. By preventing the overgrowth of pro-inflammatory pathogens, like *Porphyromonas gingivalis* and *Fusobacterium nucleatum*, it helps reduce systemic inflammation, which is a known contributor to neurodegeneration.	[190]
*Lactobacillus reuteri*	Use of mouthwash, Probiotic lozenges	*L. reuteri* has been linked to improved gut health and neurotransmitter production, particularly serotonin and oxytocin. These neurotransmitters play a crucial role in mood regulation, learning, and memory, reducing the risk of neuropsychiatric disorders, such as depression and anxiety.	[191,192]
*Bifidobacterium bifidum*	Use of mouthwash, Probiotic lozenges	This bacterium influences immune system responses, promoting an anti-inflammatory state that protects against neuroinflammation, a major contributor to neurodegenerative diseases. Individuals with cognitive impairment often have lower levels of *B. bifidum*, suggesting its potential protective role in cognitive health.	[191,192]
*Streptococcus oralis*	Typically acquired shortly after birth through interactions with caregivers and the environment	*S. oralis* contributes to the metabolism of dietary nitrates, aiding in the production of nitric oxide (NO). NO is essential for cerebrovascular health, as it promotes blood vessel dilation, enhances oxygen delivery to the brain, and reduces the risk of vascular dysfunction, which is a known contributor to cognitive decline. Poor NO metabolism has been linked to an increased risk of neurodegenerative diseases, such as Alzheimer’s disease (AD).	[193]
*Veillonella parvula*	This bacterium is a natural inhabitant of the human gut	By maintaining a balanced microbial environment in the oral cavity and gut, *V. parvula* helps prevent the overgrowth of pathogenic bacteria that contribute to systemic inflammation. Since systemic inflammation is a known driver of neuroinflammation, maintaining *V. parvula* levels may help reduce neurodegenerative risks associated with inflammatory pathways.	[79]

**Table 3:** The increase or decrease in bacteria in relation to cognitive functions within the oral–brain axis mechanism and oral–gut–brain axis mechanism.

Oral Bacterial Alteration	Oral–Brain Axis Mechanism	Oral–Gut–Brain Axis Mechanism	Reference
↑ *Porphyromonas gingivalis*	LPS crosses BBB, activating microglia and astrocytes, leading to neuroinflammation	LPS-induced gut permeability leads to systemic inflammation and neuroinflammation	[257]
↑ *Fusobacterium nucleatum*	Inflammatory cytokine release and systemic inflammation disrupt neuronal function	Translocation to the gut alters microbiota composition, impacting neurotransmitter synthesis	[258]
↑ *Treponema denticola*	Protease-induced degradation of neural and vascular tissues, exacerbating neurodegeneration	Microbial metabolites trigger immune activation, leading to chronic neuroimmune responses	[259]
↓ *Streptococcus* spp.	Reduced antimicrobial defense increases pathogenic colonization, elevating inflammatory load	Loss of butyrate-producing bacteria disrupts gut barrier integrity, increasing inflammation	[260]
↓ *Neisseria* spp.	Decreased nitric oxide production leads to cerebrovascular dysfunction	Impaired vascular function reduces oxygen delivery to the gut, affecting microbial balance	[261]
↓ *Veillonella* spp.	Altered pH and oxidative stress impair neuronal energy metabolism and synaptic function	Reduced lactate metabolism alters gut SCFA levels, impacting vagus nerve signaling	[262]
↑ *Prevotella intermedia*	Excess SCFAs lower pH, triggering neuroinflammation and oxidative stress	Excess carbohydrate fermentation alters gut microbial metabolites, influencing brain function	[72]
↑ *Tannerella forsythia*	Sialidase activity enhances immune evasion, increasing neuroinflammatory responses	Increased periodontal inflammation elevates systemic inflammatory markers, affecting gut-brain axis	[263]
↑ *Actinomyces naeslundii*	Biofilm overgrowth increases inflammation, leading to chronic neuroimmune activation	Enhanced biofilm persistence disrupts gut microbiota and affects neuroprotection	[264]

## Data Availability

No new data were created or analyzed in this study. Data sharing is not applicable to this article.
